# Combining CT Images and Clinical Features of Four Periods to Predict Whether Patients Have Rectal Cancer

**DOI:** 10.1155/2021/4662061

**Published:** 2021-12-07

**Authors:** Yingyin Feng, Qi Ding, Chen Meng, Wenfeng Wang, Jingjing Zhang, Huixiu Lian

**Affiliations:** ^1^Department of Radiology, Tianjin Fourth Central Hospital, Tianjin 300140, China; ^2^Hemodialysis Room, Tianjin Fourth Central Hospital, Tianjin 300140, China; ^3^School of Science, Shanghai Institute of Technology, Shanghai 201418, China; ^4^Department of Medical Imaging, Yantai Affiliated Hospital of Binzhou Medical University, Yantai 264100, China

## Abstract

In this paper, we mainly use random forest and broad learning system (BLS) to predict rectal cancer. A total of 246 participants with computed tomography (CT) image records were enrolled. The total model in the training set (combined with imaging and clinical indicators) has the best prediction result, with the area under the curve (AUC) of 0.999 (95% confidence internal (CI): 0.996–1.000) and the accuracy of 0.990 (95%CI: 0.976–1.000). Model 3, the general model in the test set, has the best prediction result, with the AUC of 0.962 (95%CI: 0.915–1.000) and the accuracy of 0.920 (95%CI: 0.845–0.995). The results of the model using random forest prediction are compared with those using BLS prediction. It can be found that there is no statistical difference between the two results. Our prediction model combined with image features has a good prediction result, and this image feature is the most important among all features. Consequently, we can successfully predict rectal cancer through a combination of the clinical indicators and the comprehensive indicators of CT image characteristics in four different periods (plain scan, vein, artery, and excretion).

## 1. Introduction

The incidence of rectal cancer is one of the most malignant tumors in the world [1–3]. Accurate clinical staging is the key to treatment decision, especially for rectal cancer [2, 3]. In the past century, medical imaging technology has experienced various hardships and achieved many new achievements in its continuous development [3–5]. In recent years, high resolution magnetic resonance imaging (MRI) and computed tomography (CT), along with the applications of endoscopic ultrasonography, enabled clinicians to more accurately choose corresponding treatment before surgery for rectal cancer staging, according to tumor location, infiltration depth, lymph node, and distant metastasis [1–10].

The current treatment of rectal cancer has entered into a multidisciplinary comprehensive treatment pattern [11, 12]. Among them, new adjuvant chemoradiotherapy followed by selective radical resection of rectal cancer is one of the generally recognized methods for the treatment of advanced rectal cancer [13–15]. Until now, a series of novel adjuvant chemoradiotherapy have been recommended for patients with TNM stage rectal cancer [16]. Until now, medical imaging technology is still one of the most commonly used methods to obtain noninvasive information of human tissues and organs and assist disease diagnosis, which requires to accurately predict rectal cancer with medical images and clinical features of the patients in different periods [17, 18].

The purpose of this study was to predict whether patients had rectal cancer by analyzing the CT images and clinical features over four periods. The manuscript was organized as follows. The proposed models are introduced in Section 2. In Section 3, experimental results of these models are given, and finally, the results are compared and discussed with a principle to choose the most suitable prediction model.

## 2. Proposed Models

### 2.1. Data Collection and Treatment

The data were collected from Tianjin Fourth Central Hospital from 2016 to 2021, which involve 246 individuals with CT information. All statistical tests were conducted by bilateral test [19]. During the significance test, the parameter *α* = 0.05 is employed to define whether the differences were statistically significant [20]. The measurement data were tested by Kolmogorov–Smirnov [21], where the continuous variables of normal distribution were expressed by mean standard deviation (mean ± SD). The comparison between data groups was tested by *t*-test analysis [22–24]. The measurement data of nonnormal distribution are enhanced by median and interquartile distance [25]. The rank sum test was used for comparisons among the data groups [26–28]. Classification variables will describe the number and percentage of cases of each category in  *χ*^2^ test [29]. All missing values were filled by random interpolation [30]. The gap-filling result is reliable since there is no difference before and after filling, as shown in Table 1.

**Figure 1:**
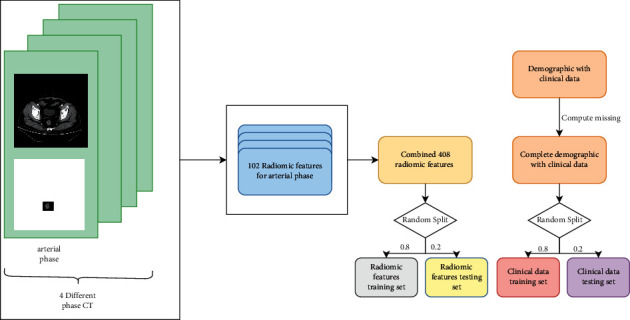
Data preprocessing process.

As seen in Figure 1, each patient has four CT images, which represent four different periods, including the plain scan period (no contrast medium was pushed), the arterial period (inject contrast agent into artery for visualization to observe whether the blood supply of diseased artery is abundant), the venous period (contrast agent enters venous blood vessels and observe the blood supply of diseased veins), and the excretion period (during which the contrast medium is excreted). The features of CT in four periods were extracted by pyradiomics and then combined with CT in four periods of a sample. The rectal part of each CT is extracted as the ROI (region of interest) [31].

Random number 2021 is used to randomly split the data into 8 : 2, 80% of the data is used as the training set to build the model, and the remaining 20% of the data is used as the verification set to verify the model. In the training set, Lasso regression (*α* = 0.01) was used to screen the clinical data and imaging features [9, 10]. The final clinical data screened out four characteristics: gender, diabetes history, family cancer history, and fecal occult blood. A total of 15 image features were screened out.

### 2.2. Construction of Prediction Model

We mainly use random forest and broad learning system (BLS) to predict rectal cancer. Broad learning maps input data and constructs the mapping features and then activates the mapping features to enhancement layers and outputs the two parts features together. In this paper, broad learning system is used to learn the variables in the model to obtain the output variables.

  Output value of mapping nodes: *Z*_*i*_=∅(*XW*_*e*_*i*__+*β*_*e*_*i*__), *i*=1,…, *n*
  Enhanced nodes output value: *H*_*j*_=*ξ*(*Z*^*n*^*W*_*h*_*j*__+*β*_*h*_*j*__), *j*=1,…, *m*
  Output nodes value: *Y*=[*Z*_1_,…, *Z*_*n*_*|ξ*(*Z*^*n*^*W*_*h*_*j*__+*β*_*h*_*j*__)]*W*^*m*^=[*Z*_1_,…, *Z*_*n*_*|H*_1_,…, *H*_*m*_]*W*^*m*^, =[*Z*^*m*^*|H*^*m*^]*W*^*m*^
  So, the pseudoinverse matrix: *W*^*m*^=[*Z*^*m*^*|H*^*m*^]^+^*Y*=(*A*^*m*^)^+^*Y*
 
$A^{+}=\underset{\lambda ⟶0}{\lim}{\left(\lambda I+AA^{T}\right)}^{-1}A^{T}$

There are *n* groups of *Z* with *k* nodes in each group and *m* groups of H with P nodes in each group.

Then, we need to screen out new features by using the loss function of the 1-norm in Lasso regression and incorporate the new features into the random forest, $J\left(w,b\right)=1/2m\underset{w,b}{\arg\min}\sum_{i=1}^{m}{\left({\hat{y}}_{i}-y_{i}\right)}^{2}+\alpha \sum_{i=1}^{n}\left\|w_{i}\right\|$. And, random forest is a combination of decision trees. Each decision tree is trained by randomly generating new data sets from the original data set. The decision result of random forest is the decision result of most decision trees. Single model classification method often has not high precision, prone to overfitting problem, so many scholars often through the combination of multiple single models to improve prediction accuracy, and these methods are called classifier combination method. Random forest is an algorithm that proposed to solve the overfitting problem of single decision tree model.

The random forest uses the Bootstrap resampling method to extract multiple samples from the original samples and then conducts decision tree modeling for each Bootstrap sample and then synthesizes multiple decision tree for prediction and obtains the final prediction result through voting. The core idea of Bootstrap resampling is to sample *n* original sample data with the sample size of *N*, and the probability of each observation object being selected is equal, that is, 1/*N*. The sample is regarded as the whole, and the subsamples sampled are regarded as samples from the sample. The resulting subsample is called the bootstrap sample.

1. Each decision tree is generated by training sample *X* with sample size *K* and random vector *θ*_*k*_
2. Random vector sequence {*θ*_*k*_, 1,…, *K*} is independently and identically distributed
3. Random forest is the set of all decision trees {*h*(*X*, *θ*_*k*_), *k*=1,2,…, *K*}

Each decision tree model *h*(*X*, *θ*_*k*_) has one vote to select the classification result of input variable *X*:

(1) $$\begin{array}{c} H\left(x\right)=\underset{Y}{\max}\sum_{i=1}^{k}I\left(h_{i}\left(x\right)=Y\right), \end{array}$$

where *H*(*x*) represents random forest classification result, *h*_*i*_(*x*) represents the classification result of a single decision tree, *Y* represents the classification target, *I*(·)  represents an indicative function, and the random forest classification model uses simple voting strategy to complete the final classification.

**Figure 2:**
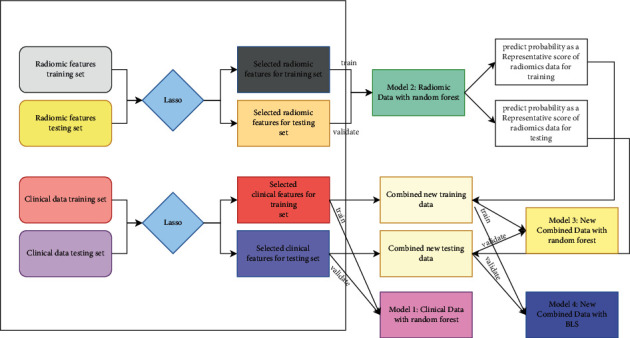
Flow chart of the prediction model development and validation.

Four models are constructed, as shown in Figure 2. In model 1, only four clinical features are used to build a prediction model with random forest. Model 2 is a prediction model based on 15 image features and random forest, whose prediction probability is also used in model 3 as a new index, combining with four clinical characteristics, using a prediction model built by random forest. Model 4 is a prediction model based on BLS training using the data of model 3.

The area under the curve (AUC) and accuracy were used to evaluate predictive performance of the models. Delong test was used to compare the evaluation indexes of the models. With *P* < 0.05, the difference was statistically significant. In this study, R (version 4.0.3) is used for clinical data preprocessing, SAS (version 9.4) is used for comparison among data groups, and Python (version 3.7.4) is used for data screening and model building.

## 3. Results and Discussion

### 3.1. Characteristics between the Training and Testing Sets

After splitting the data into the training set and the test set, the balance test is carried out, and the final *P* values of all variables are >0.05, indicating that the balance of the two groups of data is comparable. See, for details, Table 2.

### 3.2. Characteristics between Rectal Cancer and Non-Rectal Cancer Groups

Table 3 shows the characteristics of the participants between rectal cancer and non-rectal cancer groups. We can find that there are significant differences between rectal cancer and non-rectal cancer in gender, past diabetes history, family cancer history, drinking history, fecal occult blood test, carcinoembryonic antigen, and carbohydrate antigen [11, 12].

### 3.3. Comparison for the Predictive Performance of Models (1, 2, and 3)

After Delong test, it is finally found that the total model in the training set, namely, model 3, has the best prediction efficacy, with the AUC of 0.999 (95%CI: 0.996–1.000) and the accuracy of 0.990 (95%CI: 0.976–1.000). See, for details, Table 4.

**Figure 3:**
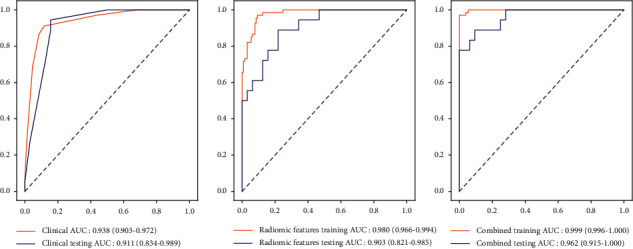
ROC curves of the prediction models.

After Delong test, the total model in the testing set, that is, model 3, has the best prediction efficacy, with the AUC of 0.962 (95%CI: 0.915–1.000) and the accuracy of 0.920 (95%CI: 0.845–0.995). See, for details, Table 5. The ROC curves of these models (1, 2, and 3) are shown in Figure 3.

### 3.4. Comparison for the Predictive Performance between Model 3 and BLS Model

**Figure 4:**
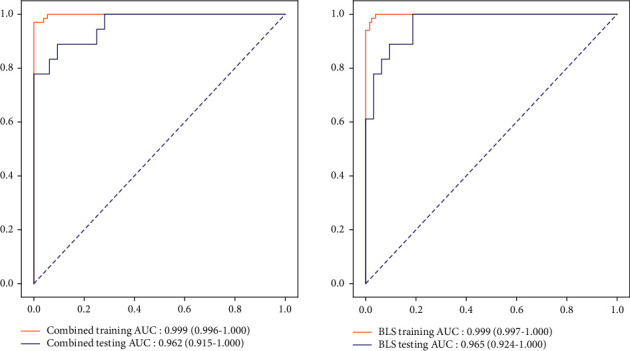
ROC curves of the prediction models.

The results of the model using the random forest analysis are compared with those using BLS prediction. It is found that there is no statistical difference between the two results, see Tables 6 and 7. The ROC curves of model 3 and BLS model are shown in Figure 4.

### 3.5. Discussion on the Importance of Variables

**Figure 5:**
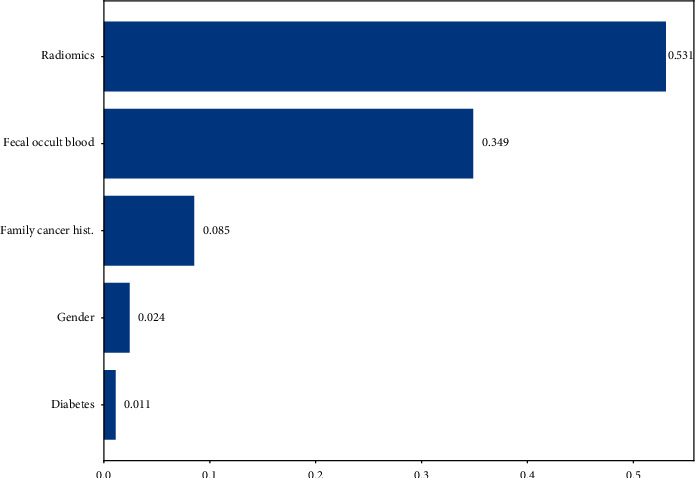
The importance of variables.

Because the prediction model established by using random forest is more interpretable, we finally choose random forest model. Finally, the importance of the characteristics of the stochastic forest model shows that our comprehensive image index score is the most important [13, 14], followed by fecal occult blood, family cancer history, gender, and diabetes. See details in Figure 5.

According to relevant studies, the early symptoms of rectal cancer are not obvious, and most patients have already developed to the stage of local progression when they visit the doctor. The ideal treatment effect cannot be achieved solely by relying on surgical treatment, and the postoperative 5-year survival rate is only about 50% [19, 20]. Result and analysis become very important to the diagnosis of rectal cancer, and foreign-related research [21] have shown that using the method of gas injection and waterflooding in rectum CT scan can find early lesions, can improve the diagnostic accuracy rate to 86%–90%, can stage judgment accuracy up to 84%, and for staging the identification of main basis insufflate the surrounding fat clearance is clear. Whether the gap between bowel and surrounding organs disappears, this will also cause a certain error. In this study, four models were mainly used for comparative analysis and intergroup comparison of relevant factors. Finally, it was found that the prediction correlation of model 3 was the best, that is, the prediction model using random forest and model 2 combined with four clinical features had the best effect. The prediction is 99 percent accurate. This paper also explored the use of random forest for model prediction after adopting the BLS learning feature. It can be found that the prediction effect of the training set is much better than that of model 3, but the test effect is still not as good as that of model 3. In summary, clinical indicators were combined with a comprehensive index of CT image features at four different periods (plain, venous, arterial, and excretory) to predict rectal cancer.

## 4. Conclusion

In this paper, after dividing the data set, we perform a balance test and can get detailed values, which can be found in Table 2. At the same time, through intergroup comparison, we can clearly find differences and their statistical significance in several aspects such as gender, previous history of diabetes, and family cancer history. Herein, four models were developed to predict the risk of rectal cancer. Our findings showed that the prediction model (model 3) which included clinical characteristics and CT images had good predictive performance for rectal cancer. It is beneficial for clinicians to identify rectal cancer cases and to improve the prognosis by early treatment.

## Figures and Tables

**Table 1:** Sensitivity analysis before/after gap-filling

Variables	Missing value	After (*n* = 246)	Before (*n* = 246)	Statistics	*P*
Total cholesterol	11 (4.47%)	4.52 ± 1.28	4.51 ± 1.30	*t* = 0.03	0.978
Triglyceride	11 (4.47%)	1.30 (0.98, 1.76)	1.30 (0.97, 1.76)	*Z* = −0.062	0.950
Lipoprotein cholesterol (LD)	12 (4.88%)	2.91 (2.30, 3.56)	2.91 (2.25, 3.60)	*Z* = −0.046	0.963
Lipoprotein cholesterol (HD)	12 (4.88%)	1.04 (0.86, 1.26)	1.04 (0.83, 1.26)	*Z* = −0.187	0.852
Carcinoembryonic antigen	3 (1.22%)	2.66 (1.81, 5.29)	2.65 (1.74, 5.17)	*Z* = −0.100	0.921
Alpha fetoprotein	6 (2.43%)	2.53 (1.88, 3.68)	2.50 (1.87, 3.66)	*Z* = −0.243	0.808
Sugar antigen 199	13 (5.28%)	9.88 (2.78, 22.28)	9.73 (2.51, 22.28)	*Z* = −0.227	0.820

LD = low density, HD = high density.

**Table 2:** Comparison for the characteristics between the training and testing sets.

Variables	Total (*n* = 246)	Training set (*n* = 196)	Testing set (*n* = 50)	Statistics	*P*
Gender, *n* (%)				*χ* ^2^ = 1.597	0.206
Male	143 (58.13)	110 (56.12)	33 (66.00)		
Female	103 (41.87)	86 (43.88)	17 (34.00)		
Past history of hypertension, *n* (%)				*χ* ^2^ = 0.194	0.659
No	126 (51.22)	99 (50.51)	27 (54.00)		
Yes	120 (48.78)	97 (49.49)	23 (46.00)		
Past history of diabetes, *n* (%)				*χ* ^2^ = 0.049	0.825
No	174 (70.73)	138 (70.41)	36 (72.00)		
Yes	72 (29.27)	58 (29.59)	14 (28.00)		
Family cancer history, *n* (%)				Fisher	0.208
No	229 (93.09)	180 (91.84)	49 (98.00)		
Yes	17 (6.91)	16 (8.16)	1 (2.00)		
History of intestinal inflammatory diseases, *n* (%)				Fisher	1.000
No	244 (99.19)	194 (98.98)	50 (100.00)		
Yes	2 (0.81)	2 (1.02)	0 (0.00)		
Smoking history, *n* (%)				*χ* ^2^ = 5.059	0.080
Never smoking	145 (58.94)	121 (61.73)	24 (48.00)		
Smoking	80 (32.52)	62 (31.63)	18 (36.00)		
Quit smoking	21 (8.54)	13 (6.63)	8 (16.00)		
Drinking history, *n* (%)				*χ* ^2^ = 2.351	0.309
Never drinking	188 (76.42)	154 (78.57)	34 (68.00)		
Drinking	51 (20.73)	37 (18.88)	14 (28.00)		
Quit drinking	7 (2.85)	5 (2.55)	2 (4.00)		
Hemoglobin, Mean ± SD	126.62 ± 28.66	125.71 ± 28.25	130.20 ± 30.24	*t* = 0.99	0.324
Total cholesterol, mean ± SD	4.52 ± 1.28	4.50 ± 1.27	4.59 ± 1.34	*t* = 0.47	0.637
Triglycerides, M (*Q*_1_, *Q*_3_)	1.30 (0.98, 1.76)	1.29 (0.97, 1.70)	1.38 (1.01, 1.94)	*Z* = 0.952	0.341
Low density lipoprotein, M (*Q*_1_, *Q*_3_)	2.91 (2.30, 3.56)	2.89 (2.32, 3.56)	2.99 (2.09, 3.79)	*Z* = 0.092	0.926
High density lipoprotein, M (*Q*_1_, *Q*_3_)	1.04 (0.86, 1.26)	1.04 (0.86, 1.26)	1.03 (0.87, 1.27)	*Z* = -0.049	0.961
Fecal occult blood test, *n* (%)				*χ* ^2^ = 0.454	0.501
No	148 (60.16)	120 (61.22)	28 (56.00)		
Yes	98 (39.84)	76 (38.78)	22 (44.00)		
Carcinoembryonic antigen, M (*Q*_1_, *Q*_3_)	2.66 (1.81, 5.29)	2.66 (1.88, 5.17)	2.65 (1.55, 7.01)	*Z* = -0.483	0.629
Alpha-fetoprotein, M (*Q*_1_, *Q*_3_)	2.53 (1.88, 3.68)	2.51 (1.88, 3.70)	2.57 (1.88, 3.32)	*Z* = -0.354	0.723
Saccharide antigen 199, M (*Q*_1_, *Q*_3_)	9.88 (2.78, 22.28)	11.28 (2.81, 23.14)	7.34 (2.51, 20.89)	*Z* = -1.204	0.229
Rectal cancer, *n* (%)				*χ* ^2^ = 0.058	0.809
No	161 (65.45)	129 (65.82)	32 (64.00)		
Yes	85 (34.55)	67 (34.18)	18 (36.00)		

**Table 3:** Comparison for the characteristics between rectal cancer and non-rectal cancer groups.

Variables	Total (*n* = 196)	Non-rectal cancer (*n* = 129)	Rectal cancer (*n* = 67)	Statistics	*P*
Gender, *n* (%)				*χ* ^2^ = 9.957	0.002
Male	110 (56.12)	62 (48.06)	48 (71.64)		
Female	86 (43.88)	67 (51.94)	19 (28.36)		
Past history of hypertension, *n* (%)				*χ* ^2^ = 0.905	0.341
No	99 (50.51)	62 (48.06)	37 (55.22)		
Yes	97 (49.49)	67 (51.94)	30 (44.78)		
Past history of diabetes, *n* (%)				*χ* ^2^ = 6.667	0.010
No	138 (70.41)	83 (64.34)	55 (82.09)		
Yes	58 (29.59)	46 (35.66)	12 (17.91)		
Family cancer history, *n* (%)				*χ* ^2^ = 27.476	<0.001
No	180 (91.84)	128 (99.22)	52 (77.61)		
Yes	16 (8.16)	1 (0.78)	15 (22.39)		
History of intestinal inflammatory diseases, *n* (%)				Fisher	0.548
No	194 (98.98)	127 (98.45)	67 (100.00)		
Yes	2 (1.02)	2 (1.55)	0 (0.00)		
Smoking history, *n* (%)				*χ* ^2^ = 1.384	0.501
Never smoking	121 (61.73)	81 (62.79)	40 (59.70)		
Smoking	62 (31.63)	38 (29.46)	24 (35.82)		
Quit smoking	13 (6.63)	10 (7.75)	3 (4.48)		
Drinking history, *n* (%)				Fisher	0.044
Never drinking	154 (78.57)	107 (82.95)	47 (70.15)		
Drinking	37 (18.88)	18 (13.95)	19 (28.36)		
Quit drinking	5 (2.55)	4 (3.10)	1 (1.49)		
Hemoglobin, mean ± SD	125.71 ± 28.25	124.85 ± 29.84	127.36 ± 25.05	*t* = −0.59	0.557
Total cholesterol, mean ± SD	4.50 ± 1.27	4.54 ± 1.34	4.42 ± 1.12	*t* = 0.63	0.530
Triglycerides, M (*Q*_1_, *Q*_3_)	1.29 (0.97, 1.70)	1.35 (0.96, 1.86)	1.21 (1.00, 1.56)	*Z* = −1.424	0.154
Low density lipoprotein, M (*Q*_1_, *Q*_3_)	2.89 (2.32, 3.56)	2.90 (2.26, 3.52)	2.88 (2.38, 3.56)	*Z* = 0.451	0.652
High density lipoprotein, M (*Q*_1_, *Q*_3_)	1.04 (0.86, 1.26)	1.02 (0.81, 1.22)	1.10 (0.91, 1.26)	*Z* = 1.499	0.134
Fecal occult blood test, *n* (%)				*χ* ^2^ = 117.152	<0.001
No	120 (61.22)	114 (88.37)	6 (8.96)		
Yes	76 (38.78)	15 (11.63)	61 (91.04)		
Carcinoembryonic antigen, M (*Q*_1_, *Q*_3_)	2.66 (1.88, 5.17)	2.45 (1.71, 3.93)	4.71 (2.54, 20.24)	*Z* = 5.515	<0.001
Alpha-fetoprotein, M (*Q*_1_, *Q*_3_)	2.51 (1.88, 3.70)	2.44 (1.77, 3.68)	2.73 (2.04, 3.74)	*Z* = 1.006	0.314
Saccharide antigen 199, M (*Q*_1_, *Q*_3_)	11.28 (2.81, 23.14)	9.30 (0.97, 17.38)	19.85 (6.57, 58.40)	*Z* = 4.294	<0.001

**Table 4:** The predictive performance of the prediction models using the training set.

Variables	Clinical demographics	Radiomic	Total model
Cut off	0.426	0.265	0.557
Sensitivity (95% CI)	0.910 (0.842–0.979)	0.970 (0.929–1.000)	0.970 (0.929–1.000)
Specificity (95% CI)	0.884 (0.828–0.939)^*∗*^	0.907 (0.857–0.957)^*∗*^	1.000 (1.000–1.000)
PPV (95% CI)	0.803 (0.713–0.892)^*∗*^	0.844 (0.763–0.925)^*∗*^	1.000 (1.000–1.000)
NPV (95% CI)	0.950 (0.911–0.989)	0.983 (0.960–1.000)	0.985 (0.964–1.000)
AUC (95% CI)	0.938 (0.903–0.972)^*∗*^	0.980 (0.966–0.994)	0.999 (0.996–1.000)
Accuracy (95% CI)	0.893 (0.850–0.936)^*∗*^	0.929 (0.893–0.965)^*∗*^	0.990 (0.976–1.000)

^*∗*^Compared with the total model, the difference is statistically significant. CI: confidence interval; PPV: positive predictive value; NPV: negative predictive value; AUC: area under the curve.

**Table 5:** The predictive performance of the prediction models using the testing set.

Variables	Clinical demographics	Radiomic	Total model
Cut off	0.426	0.265	0.557
Sensitivity (95% CI)	0.944 (0.839–1.000)^*∗*^	0.778 (0.586–0.970)	0.778 (0.586–0.970)
Specificity (95% CI)	0.844 (0.718–0.970)^*∗*^	0.844 (0.718–0.970)	1.000 (1.000–1.000)
PPV (95% CI)	0.773 (0.598–0.948)^*∗*^	0.737 (0.539–0.935)^*∗*^	1.000 (1.000–1.000)
NPV (95% CI)	0.964 (0.896–1.000)^*∗*^	0.871 (0.753–0.989)	0.889 (0.786–0.992)
AUC (95% CI)	0.911 (0.834–0.989)	0.903 (0.821–0.985)	0.962 (0.915–1.000)
Accuracy (95% CI)	0.880 (0.790–0.970)	0.820 (0.714–0.926)	0.920 (0.845–0.995)

CI: confidence interval; PPV: positive predictive value; NPV: negative predictive value; AUC: area under the curve.

**Table 6:** The predictive performance of the prediction models using the training set.

Variables	Total model	BLS model
Cutoff	0.557	0.461
Sensitivity (95% CI)	0.970 (0.929–1.000)	0.985 (0.956–1.000)
Specificity (95% CI)	1.000 (1.000–1.000)	0.977 (0.951–1.000)
PPV (95% CI)	1.000 (1.000–1.000)	0.957 (0.908–1.000)
NPV (95% CI)	0.985 (0.964–1.000)	0.992 (0.977–1.000)
AUC (95% CI)	0.999 (0.996–1.000)	0.999 (0.997–1.000)
Accuracy (95% CI)	0.990 (0.976–1.000)	0.980 (0.960–0.999)

CI: confidence interval; PPV: positive predictive value; NPV: negative predictive value; AUC: area under the curve.

**Table 7:** The predictive performance of the prediction models using the testing set.

Variables	Total model	BLS model
Sensitivity (95% CI)	0.778 (0.586–0.970)	0.889 (0.744–1.000)
Specificity (95% CI)	1.000 (1.000–1.000)	0.906 (0.805–1.000)
PPV (95% CI)	1.000 (1.000–1.000)	0.842 (0.678–1.000)
NPV (95% CI)	0.889 (0.786–0.992)	0.935 (0.849–1.000)
AUC (95% CI)	0.962 (0.915–1.000)	0.965 (0.924–1.000)
Accuracy (95% CI)	0.920 (0.845–0.995)	0.900 (0.817–0.983)

CI: confidence interval; PPV: positive predictive value; NPV: negative predictive value; AUC: area under the curve.

## Data Availability

The data utilized to support the findings are available from the corresponding authors upon request.

## References

[1] Sartori A., Souza A., Zanon M. (2017). Performance of magnetic resonance imaging in pulmonary fungal disease compared to high‐resolution computed tomography. *Mycoses*.

[2] Counter S. A., Damberg P., Aski S. N., Engmér Berglin C., Laurell G. (2015). Experimental fusion of contrast enhanced high-field magnetic resonance imaging and high-resolution micro-computed tomography in imaging the mouse inner ear. *The Open Neuroimaging Journal*.

[3] Rollvén E., Holm T., Glimelius B., Lörinc E., Blomqvist L. (2013). Potentials of high resolution magnetic resonance imaging versus computed tomography for preoperative local staging of colon cancer. *Acta Radiologica*.

[4] Kolk A., Stimmer H., Klopfer M. (2009). High resolution magnetic resonance imaging with an orbital coil as an alternative to computed tomography scan as the primary imaging modality of pediatric orbital fractures. *Journal of Oral and Maxillofacial Surgery*.

[5] Fink C., Grenacher L., Hansmann H. J. (2001). Prospektive Studie zum Vergleich der hochauflösenden Computertomographie und Magnetresonanztomographie in der Detektion von Pankreasneoplasien: verwendung intravenöser und oraler MR-Kontrastmittel. *RöFo - Fortschritte auf dem Gebiet der Röntgenstrahlen und der bildgebenden Verfahren*.

[6] Hamamichi Y., Ichida F., Hashimoto I. (2001). Isolated noncompaction of the ventricular myocardium: ultrafast computed tomography and magnetic resonance imaging. *The International Journal of Cardiac Imaging*.

[7] Kolk A., Pautke C., Schott V. (2007). Secondary post-traumatic enophthalmos: high-resolution magnetic resonance imaging compared with multislice computed tomography in postoperative orbital volume measurement. *Journal of Oral and Maxillofacial Surgery*.

[8] Stimmer H., Arnold W., Schwaiger M., Laubenbacher C. (2002). Magnetic resonance imaging and high-resolution computed tomography in the otospongiotic phase of otosclerosis. *ORL*.

[9] Buch K., Morancy T., Kaplan I. (2015). Improved dosimetry in prostate brachytherapy using high resolution contrast enhanced magnetic resonance imaging: a feasibility study. *Journal of Contemporary Brachytherapy*.

[10] Schmidt G. P., Baurmelnyk A., Herzog P. (2005). High-resolution whole-body magnetic resonance image tumor staging with the use of parallel imaging versus dual-modality positron emission tomography-computed tomography: experience on a 32-channel system. *Investigative Radiology*.

[11] Bianchi P., Ceriani C., Rottoli M. (2005). Endoscopic ultrasonography and magnetic resonance in preoperative staging of rectal cancer: comparison with histologic findings. *Journal of Gastrointestinal Surgery*.

[12] Inoue Y., Murata Y., Suzuki M. (2011). Analysis of distant metastasis of rectal cancer using color Doppler endoscopic ultrasonography. *Gastroenterological Endoscopy*.

[13] Chessin D. B., Enker W., Cohen A. M. (2005). Complications after preoperative combined modality therapy and radical resection of locally advanced rectal cancer: a 14-year experience from a specialty service. *Journal of the American College of Surgeons*.

[14] Camp E. R., Lucas J. T., Simpson K., Esnaola N. F. (2011). Local excision and selective radical resection after neoadjuvant chemoradiation for rectal cancer. *Journal of Clinical Oncology*.

[15] Takeuchi Y., Minami S., Sugino S. (2009). A case of carcinoma in adenoma arising in artificial anus twelve years after radical resection of rectal cancer. *Nihon Rinsho Geka Gakkai Zasshi*.

[16] Bosset J.-F., Calais G., Mineur L. (2014). Fluorouracil-based adjuvant chemotherapy after preoperative chemoradiotherapy in rectal cancer: long-term results of the EORTC 22921 randomised study. *The Lancet Oncology*.

[17] Lee D. J.-K., Sagar P. M., Sadadcharam G., Tan K.-Y. (2017). Advances in surgical management for locally recurrent rectal cancer: how far have we come?. *World Journal of Gastroenterology*.

[18] Wei D. (2018). Progress in prevention and treatment of anastomotic leakage after surgery for rectal cancer. *World Chinese Journal of Digestology*.

[19] Hahn P., Wolf M. B., Unglaub F. (2013). Bilateral test for potential subluxation of the DRUJ. *Archives of Orthopaedic and Trauma Surgery*.

[20] Hatipoglu H., Canbaz Kabay S., Gungor Hatipoglu M., Ozden H. (2015). Expanded disability status scale-based disability and dental-periodontal conditions in patients with multiple sclerosis. *Medical Principles and Practice*.

[21] Japar O. A., Evardone C. P. (2019). Comparison of bootstrap-estimated and half sample-estimated Kolmogorov-smirnov test statistics. *European Academic Research*.

[22] Ruxton G. D. (2010). The unequal variance t-test is an underused alternative to Student’s t-test and the Mann–Whitney U test. *Behavioral Ecology*.

[23] Chaves R., Ramírez J., Górriz J. M. (2009). SVM-based computer-aided diagnosis of the Alzheimer’s disease using t-test NMSE feature selection with feature correlation weighting. *Neuroscience Letters*.

[24] Fuse H., Kazama T., Katayama T. (1991). Relationship between hypoosmotic swelling test, semen analysis, and zona-free hamster ovum test. *Archives of Andrology*.

[25] Scollar I., Weidner B., Huang T. S. (1984). Image enhancement using the median and the interquartile distance. *Computer Vision, Graphics, and Image Processing*.

[26] Hallstrom A. P. (1989). A “weighted” rank sum test. *Controlled Clinical Trials*.

[27] Matsumoto S., Murakami N., Koizumi H., Takahashi M., Izumi Y., Kaji R. (2017). Evaluation of the edrophonium challenge test for cervical dystonia. *Internal Medicine*.

[28] Placencio-Hickok V., Lauzon M., Moshayedi N. (2021). Hyaluronan heterogeneity in pancreatic ductal adenocarcinoma, primary tumors, and sites of metastasis. *Journal of Clinical Oncology*.

[29] McHugh M. L. (2013). The Chi-square test of independence. *Biochemia Medica*.

[30] Yang Y., Wei Y. S. (2013). Random interpolation average for ECG signal denoising using multiple wavelet bases. *Biomedical Engineering Applications Basis and Communications*.

[31] Yamada N., Kubo M., Kawata Y. (2003). ROI extraction of chest CT images using adaptive opening filter. *Proceedings of SPIE—The International Society for Optical Engineering*.

